# Crystal structure of methyl 3-(3-fluoro­phen­yl)-1-methyl-1,3a,4,9b-tetra­hydro-3*H*-thio­chromeno[4,3-*c*]isoxazole-3a-carboxyl­ate

**DOI:** 10.1107/S2056989015013651

**Published:** 2015-07-25

**Authors:** M. P. Savithri, M. Suresh, R. Raghunathan, G. Vimala, A. SubbiahPandi

**Affiliations:** a Department of Physics, Queen Mary’s College (Autonomous), Chennai 600 004, India; b Department of Organic Chemistry, University of Madras, Guindy Campus, Chennai 600 025, India; c Department of Physics, Presidency College (Autonomous), Chennai 600 005, India

**Keywords:** crystal structure, oxazolidine, thio­pyran, thio­chromenone, C—H⋯π inter­actions

## Abstract

In the title compound, C_19_H_18_FNO_3_S, the five-membered oxazolidine ring adopts an envelope conformation with the methine C atom of the fused bond as the flap. Its mean plane is oriented at a dihedral angle of 50.38 (1)° with respect to the fluoro­phenyl ring. The six-membered thio­pyran ring has a half-chair conformation and its mean plane is almost coplanar with the fused benzene ring, making a dihedral angle of 4.94 (10)°. The two aromatic rings are inclined to one another by 85.96 (11)°, and the mean planes of the oxazolidine and thio­pyran rings are inclined to one another by 57.64 (12)°. In the crystal, mol­ecules are linked by C—H⋯π inter­actions, forming a three-dimensional structure.

## Related literature   

For background on thio-containing heterocyclic rings and for related structures, see for example: Khan *et al.* (2008*a* ▸,*b* ▸).
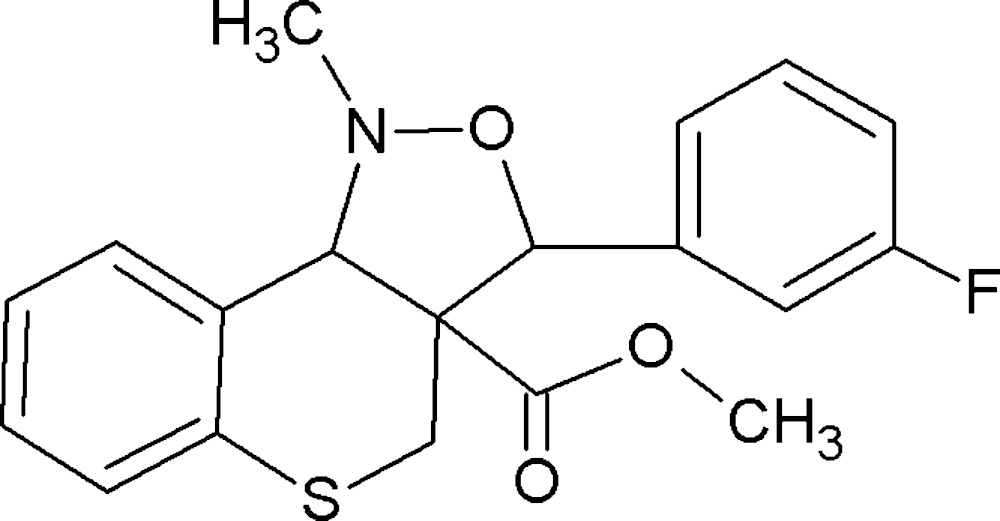


## Experimental   

### Crystal data  

C_19_H_18_FNO_3_S
*M* *_r_* = 359.40
Monoclinic, 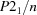

*a* = 10.7729 (8) Å
*b* = 12.6361 (8) Å
*c* = 12.625 (1) Å
β = 92.992 (3)°
*V* = 1716.3 (2) Å^3^
*Z* = 4
Mo *K*α radiation
μ = 0.22 mm^−1^
*T* = 293 K
0.30 × 0.30 × 0.25 mm

### Data collection  

Bruker Kappa APEXII CCD diffractometer
Absorption correction: multi-scan (*SADABS*; Bruker, 2004 ▸) *T*_min_ = 0.938, *T*_max_ = 0.948
18645 measured reflections
3024 independent reflections
2456 reflections with *I* > 2σ(*I*)
*R*_int_ = 0.026

### Refinement  

*R*[*F* ^2^ > 2σ(*F* ^2^)] = 0.041
*wR*(*F* ^2^) = 0.123
*S* = 0.99
3024 reflections
243 parameters
H atoms treated by a mixture of independent and constrained refinement
Δρ_max_ = 0.52 e Å^−3^
Δρ_min_ = −0.24 e Å^−3^

### 

Data collection: *APEX2* (Bruker, 2004 ▸); cell refinement: *APEX2* and *SAINT* (Bruker, 2004 ▸); data reduction: *SAINT* and *XPREP* (Bruker, 2004 ▸); program(s) used to solve structure: *SHELXS97* (Sheldrick, 2008 ▸); program(s) used to refine structure: *SHELXL97* (Sheldrick, 2008 ▸); molecular graphics: *ORTEP-3 for Windows* (Farrugia, 2012 ▸); software used to prepare material for publication: *SHELXL97* and *PLATON* (Spek, 2009 ▸).

## Supplementary Material

Crystal structure: contains datablock(s) global, I. DOI: 10.1107/S2056989015013651/su5169sup1.cif

Structure factors: contains datablock(s) I. DOI: 10.1107/S2056989015013651/su5169Isup2.hkl

Supporting information file. DOI: 10.1107/S2056989015013651/su5169Isup3.cml

**Fig. 1.**
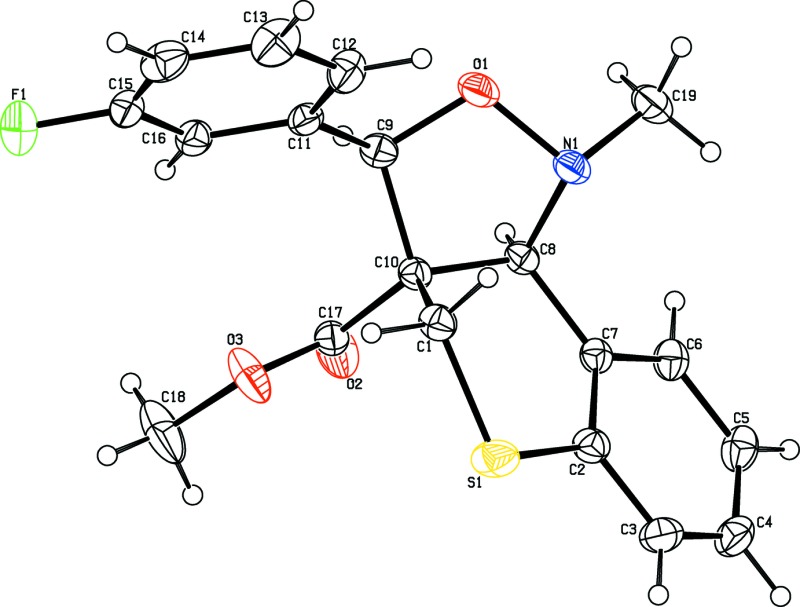
The molecular structure of the title compound, with the atom labelling. Displacement ellipsoids are drawn at the 30% probability level.

. DOI: 10.1107/S2056989015013651/su5169fig1.tif
The mol­ecular structure of the title compound, with the atom labelling. Displacement ellipsoids are drawn at the 30% probability level.

CCDC reference: 1413525

Additional supporting information:  crystallographic information; 3D view; checkCIF report

## Figures and Tables

**Table 1:** Hydrogen-bond geometry (, ) *Cg*3 and *Cg*4 are the centroids of rings C2C7 and C11C16, respectively.

*D*H*A*	*D*H	H*A*	*D* *A*	*D*H*A*
C6H6*Cg*4^i^	0.93	2.75	3.479(3)	136
C13H13*Cg*3^ii^	0.93	2.74	3.599(3)	153

Symmetry codes: (i) 
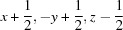
; (ii) 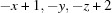.

## supplementary crystallographic information

### S1. Structural commentary

Small substituted heterocyclic compounds play an important role in the development of biologically active substances by offering a high structural diversity. In view of their biological importance, the title compound was synthesized and we report herein on its crystal structure.

The molecular structure of the title compound is illustrated in Fig. 1. The five-membered oxazolidine ring [O1/N1/C8—C10] exhibits an envelope conformation with atom C8 as the flap [asymmetry parameter ΔCs(C8) = 2.6 (2)° and puckering parameters of q_2_ = 0.483 (2)Å and φ_2_ = 256.6 (3)°]. Its mean pane is oriented at a dihedral angle of 50.38 (13)° with respect to the fluoro­phenyl ring (C11—C16). The six membered thio­pyran ring (S1/C1/C2/C7/C8/C10) has a half-chair conformation and its mean plane is almost coplanar with the fused benzene ring (C2—C7) with a dihedral angle = 4.94 (10) °. This aromatic ring is almost normal to the fluoro­phenyl ring with a dihedral angle of 85.96 (11) °. The sum of angles at atom N1 of the pyrrolidine ring (320°) is in accordance with *sp*^3^ hybridization.

In the crystal, molecules are linked by C—H···π inter­actions forming a three-dimensional structure (Table 1).

The crystal structures of 7-nitro-5*H*-1-benzo­thio­pyrano[2,3-*b*]- pyridin-5-one (Khan *et al.*, 2008a) and 5*H*-1-benzo­thio­pyrano[2,3-*b*] pyridin-5-one (Khan *et al.*, 2008b), are similar to that of the title compound.

### S2. Synthesis and crystallization

To a solution of methyl (Z)-2-(((2-formyl­phenyl)­thio)­methyl)-3-phenyl­acrylate (1 mmol) and N-methyl hydroxyl­amine hydro­chloride (1.1 mmol) in aceto­nitrile (10 ml) was added pyridine (0.2 mmol). The solution was refluxed until the completion of the reaction (monitored by TLC). The solvent was then removed under vacuum. The crude product was subjected to column chromatography on silica gel (100-200 mesh) using petroleum ether-ethyl acetate (9:1) as eluent, which successfully provided the pure product as a colorless solid. The product was dissolved in chloro­form and heated for 2 min. The resulting solution was subjected to crystallization by slow evaporation of the solvent for 48 h resulting in the formation of single crystals of the title compound.

### S3. Refinement

Crystal data, data collection and structure refinement details are summarized in Table 2. The H atoms attached to atom C1 were freely refined. All other H atoms were fixed geometrically and allowed to ride on their parent atoms: C—H = 0.93–0.98 Å with *U*_iso_(H) = 1.5*U*_eq_(C) for methyl H atoms and 1.2*U*_eq_(C) for other H atoms.

### Crystal data

**Table:**

C_19_H_18_FNO_3_S	*F*(000) = 752
*M**_r_* = 359.40	*D*_x_ = 1.391 Mg m^−^^3^
Monoclinic, *P*2_1_/*n*	Mo *K*α radiation, λ = 0.71073 Å
Hall symbol: -P 2yn	Cell parameters from 3024 reflections
*a* = 10.7729 (8) Å	θ = 2.3–25.0°
*b* = 12.6361 (8) Å	µ = 0.22 mm^−^^1^
*c* = 12.625 (1) Å	*T* = 293 K
β = 92.992 (3)°	Block, colourless
*V* = 1716.3 (2) Å^3^	0.30 × 0.30 × 0.25 mm
*Z* = 4	

### Data collection

**Table:**

Bruker Kappa APEXII CCD diffractometer	3024 independent reflections
Radiation source: fine-focus sealed tube	2456 reflections with *I* > 2σ(*I*)
Graphite monochromator	*R*_int_ = 0.026
ω and φ scans	θ_max_ = 25.0°, θ_min_ = 2.3°
Absorption correction: multi-scan (*SADABS*; Bruker, 2004)	*h* = −12→12
*T*_min_ = 0.938, *T*_max_ = 0.948	*k* = −15→15
18645 measured reflections	*l* = −15→13

### Refinement

**Table:**

Refinement on *F*^2^	Secondary atom site location: difference Fourier map
Least-squares matrix: full	Hydrogen site location: inferred from neighbouring sites
*R*[*F*^2^ > 2σ(*F*^2^)] = 0.041	H atoms treated by a mixture of independent and constrained refinement
*wR*(*F*^2^) = 0.123	*w* = 1/[σ^2^(*F*_o_^2^) + (0.0547*P*)^2^ + 1.7913*P*] where *P* = (*F*_o_^2^ + 2*F*_c_^2^)/3
*S* = 0.99	(Δ/σ)_max_ < 0.001
3024 reflections	Δρ_max_ = 0.52 e Å^−^^3^
243 parameters	Δρ_min_ = −0.24 e Å^−^^3^
0 restraints	Extinction correction: *SHELXL97* (Sheldrick, 2008), Fc^*^=kFc[1+0.001xFc^2^λ^3^/sin(2θ)]^-1/4^
Primary atom site location: structure-invariant direct methods	Extinction coefficient: 0.0079 (14)

### Special details

**Table:**

Geometry. All esds (except the esd in the dihedral angle between two l.s. planes) are estimated using the full covariance matrix. The cell esds are taken into account individually in the estimation of esds in distances, angles and torsion angles; correlations between esds in cell parameters are only used when they are defined by crystal symmetry. An approximate (isotropic) treatment of cell esds is used for estimating esds involving l.s. planes.
Refinement. Refinement of F^2^ against ALL reflections. The weighted R-factor wR and goodness of fit S are based on F^2^, conventional R-factors R are based on F, with F set to zero for negative F^2^. The threshold expression of F^2^ > 2sigma(F^2^) is used only for calculating R-factors(gt) etc. and is not relevant to the choice of reflections for refinement. R-factors based on F^2^ are statistically about twice as large as those based on F, and R- factors based on ALL data will be even larger.

### Fractional atomic coordinates and isotropic or equivalent isotropic displacement parameters (Å^2^)

**Table:**

	*x*	*y*	*z*	*U*_iso_*/*U*_eq_	
S1	0.38912 (6)	−0.09256 (5)	0.73827 (6)	0.0484 (2)	
O3	0.14448 (16)	0.08198 (17)	0.72833 (14)	0.0580 (6)	
O1	0.44199 (15)	0.19965 (17)	0.95772 (13)	0.0560 (5)	
F1	−0.09742 (14)	0.14114 (17)	1.01633 (15)	0.0759 (6)	
C7	0.54244 (19)	0.08164 (16)	0.70941 (16)	0.0285 (5)	
C2	0.5127 (2)	−0.02335 (18)	0.68520 (17)	0.0344 (5)	
C10	0.34366 (18)	0.10865 (17)	0.81229 (16)	0.0288 (5)	
C9	0.3219 (2)	0.18273 (19)	0.90866 (17)	0.0342 (5)	
C8	0.47396 (19)	0.14639 (17)	0.78729 (16)	0.0285 (5)	
C17	0.25020 (19)	0.13405 (18)	0.72143 (16)	0.0331 (5)	
C11	0.2322 (2)	0.14500 (17)	0.98805 (16)	0.0314 (5)	
C1	0.3454 (2)	−0.00741 (18)	0.84444 (18)	0.0344 (5)	
C16	0.1063 (2)	0.16036 (18)	0.96619 (18)	0.0370 (5)	
H16	0.0780	0.1928	0.9034	0.044*	
C14	0.0609 (3)	0.0799 (2)	1.1315 (2)	0.0494 (7)	
H14	0.0030	0.0583	1.1791	0.059*	
C6	0.6370 (2)	0.12968 (19)	0.65535 (18)	0.0359 (5)	
H6	0.6571	0.1999	0.6700	0.043*	
C12	0.2719 (2)	0.0969 (2)	1.08214 (19)	0.0435 (6)	
H12	0.3562	0.0861	1.0977	0.052*	
C4	0.6718 (2)	−0.0280 (2)	0.55821 (19)	0.0455 (6)	
H4	0.7149	−0.0649	0.5081	0.055*	
C5	0.7016 (2)	0.0757 (2)	0.58066 (19)	0.0426 (6)	
H5	0.7647	0.1091	0.5459	0.051*	
C3	0.5789 (2)	−0.0770 (2)	0.60926 (19)	0.0442 (6)	
H3	0.5594	−0.1472	0.5933	0.053*	
C15	0.0242 (2)	0.1273 (2)	1.0381 (2)	0.0443 (6)	
C13	0.1858 (3)	0.0649 (2)	1.1532 (2)	0.0525 (7)	
H13	0.2130	0.0328	1.2165	0.063*	
C19	0.6429 (2)	0.2148 (2)	0.9055 (2)	0.0502 (7)	
H19A	0.6781	0.2105	0.9768	0.075*	
H19B	0.6183	0.2864	0.8902	0.075*	
H19C	0.7036	0.1929	0.8570	0.075*	
C18	0.0474 (3)	0.1068 (3)	0.6484 (3)	0.0771 (11)	
H18A	−0.0251	0.0652	0.6606	0.116*	
H18B	0.0759	0.0911	0.5794	0.116*	
H18C	0.0270	0.1806	0.6524	0.116*	
N1	0.53515 (17)	0.14612 (15)	0.89425 (14)	0.0357 (5)	
O2	0.26780 (16)	0.19605 (16)	0.65350 (14)	0.0534 (5)	
H1B	0.264 (2)	−0.0329 (19)	0.8609 (18)	0.038 (6)*	
H1A	0.403 (2)	−0.0170 (19)	0.902 (2)	0.039 (6)*	
H8	0.469 (2)	0.2167 (19)	0.7624 (17)	0.030 (6)*	
H9	0.294 (2)	0.249 (2)	0.8768 (19)	0.037 (6)*	

### Atomic displacement parameters (Å^2^)

**Table:**

	*U*^11^	*U*^22^	*U*^33^	*U*^12^	*U*^13^	*U*^23^
S1	0.0561 (4)	0.0372 (4)	0.0531 (4)	−0.0166 (3)	0.0129 (3)	−0.0116 (3)
O3	0.0359 (9)	0.0920 (15)	0.0446 (10)	−0.0205 (9)	−0.0129 (8)	0.0205 (10)
O1	0.0364 (9)	0.0935 (15)	0.0382 (10)	−0.0165 (9)	0.0046 (7)	−0.0286 (10)
F1	0.0360 (9)	0.1030 (15)	0.0885 (13)	0.0088 (9)	0.0031 (8)	−0.0189 (11)
C7	0.0278 (10)	0.0316 (11)	0.0257 (10)	0.0021 (8)	−0.0016 (8)	0.0039 (8)
C2	0.0364 (12)	0.0367 (12)	0.0299 (11)	0.0004 (10)	−0.0013 (9)	−0.0009 (9)
C10	0.0277 (11)	0.0351 (11)	0.0234 (10)	−0.0018 (9)	0.0002 (8)	0.0002 (9)
C9	0.0367 (12)	0.0381 (13)	0.0276 (11)	0.0003 (10)	−0.0007 (9)	−0.0011 (10)
C8	0.0299 (11)	0.0270 (11)	0.0283 (11)	−0.0015 (8)	−0.0019 (9)	0.0013 (9)
C17	0.0294 (11)	0.0443 (13)	0.0255 (11)	0.0005 (9)	0.0013 (9)	−0.0022 (10)
C11	0.0343 (11)	0.0336 (11)	0.0263 (11)	0.0049 (9)	0.0027 (9)	−0.0032 (9)
C1	0.0346 (12)	0.0361 (12)	0.0325 (12)	−0.0065 (10)	0.0030 (10)	0.0011 (10)
C16	0.0376 (12)	0.0389 (13)	0.0342 (12)	0.0084 (10)	−0.0017 (10)	−0.0039 (10)
C14	0.0585 (17)	0.0457 (15)	0.0462 (15)	−0.0087 (12)	0.0220 (13)	−0.0039 (12)
C6	0.0331 (11)	0.0378 (12)	0.0368 (12)	0.0033 (9)	0.0017 (9)	0.0067 (10)
C12	0.0433 (14)	0.0528 (15)	0.0343 (13)	0.0136 (11)	0.0031 (10)	0.0062 (11)
C4	0.0452 (14)	0.0588 (16)	0.0329 (13)	0.0151 (12)	0.0067 (11)	−0.0033 (11)
C5	0.0375 (13)	0.0552 (15)	0.0358 (13)	0.0082 (11)	0.0084 (10)	0.0105 (11)
C3	0.0521 (15)	0.0423 (14)	0.0382 (13)	0.0043 (11)	0.0015 (11)	−0.0095 (11)
C15	0.0309 (12)	0.0481 (14)	0.0545 (16)	0.0000 (10)	0.0071 (11)	−0.0169 (12)
C13	0.0671 (18)	0.0536 (16)	0.0377 (14)	0.0109 (14)	0.0107 (13)	0.0133 (12)
C19	0.0375 (13)	0.0671 (18)	0.0456 (15)	−0.0126 (12)	−0.0028 (11)	−0.0141 (13)
C18	0.0421 (16)	0.127 (3)	0.0600 (19)	−0.0163 (18)	−0.0212 (14)	0.0236 (19)
N1	0.0334 (10)	0.0433 (11)	0.0299 (10)	−0.0046 (8)	−0.0039 (8)	−0.0046 (8)
O2	0.0442 (10)	0.0722 (13)	0.0432 (10)	−0.0029 (9)	−0.0048 (8)	0.0240 (9)

### Geometric parameters (Å, º)

**Table:**

S1—C2	1.755 (2)	C1—H1A	0.94 (3)
S1—C1	1.801 (2)	C16—C15	1.366 (3)
O3—C17	1.322 (3)	C16—H16	0.9300
O3—C18	1.449 (3)	C14—C15	1.363 (4)
O1—C9	1.422 (3)	C14—C13	1.372 (4)
O1—N1	1.480 (2)	C14—H14	0.9300
F1—C15	1.336 (3)	C6—C5	1.381 (3)
C7—C6	1.395 (3)	C6—H6	0.9300
C7—C2	1.395 (3)	C12—C13	1.383 (4)
C7—C8	1.502 (3)	C12—H12	0.9300
C2—C3	1.400 (3)	C4—C3	1.367 (4)
C10—C17	1.520 (3)	C4—C5	1.375 (4)
C10—C1	1.522 (3)	C4—H4	0.9300
C10—C8	1.531 (3)	C5—H5	0.9300
C10—C9	1.562 (3)	C3—H3	0.9300
C9—C11	1.505 (3)	C13—H13	0.9300
C9—H9	0.97 (3)	C19—N1	1.450 (3)
C8—N1	1.471 (3)	C19—H19A	0.9600
C8—H8	0.94 (2)	C19—H19B	0.9600
C17—O2	1.184 (3)	C19—H19C	0.9600
C11—C12	1.382 (3)	C18—H18A	0.9600
C11—C16	1.384 (3)	C18—H18B	0.9600
C1—H1B	0.97 (2)	C18—H18C	0.9600
			
C2—S1—C1	102.68 (11)	C11—C16—H16	120.4
C17—O3—C18	116.1 (2)	C15—C14—C13	118.1 (2)
C9—O1—N1	108.84 (15)	C15—C14—H14	121.0
C6—C7—C2	118.2 (2)	C13—C14—H14	121.0
C6—C7—C8	118.65 (19)	C5—C6—C7	121.7 (2)
C2—C7—C8	123.12 (19)	C5—C6—H6	119.1
C7—C2—C3	119.4 (2)	C7—C6—H6	119.1
C7—C2—S1	124.10 (17)	C11—C12—C13	119.9 (2)
C3—C2—S1	116.34 (18)	C11—C12—H12	120.1
C17—C10—C1	113.77 (18)	C13—C12—H12	120.1
C17—C10—C8	110.91 (17)	C3—C4—C5	120.3 (2)
C1—C10—C8	110.89 (18)	C3—C4—H4	119.9
C17—C10—C9	109.91 (17)	C5—C4—H4	119.9
C1—C10—C9	111.71 (17)	C4—C5—C6	119.4 (2)
C8—C10—C9	98.67 (16)	C4—C5—H5	120.3
O1—C9—C11	111.01 (18)	C6—C5—H5	120.3
O1—C9—C10	105.01 (17)	C4—C3—C2	121.0 (2)
C11—C9—C10	117.17 (19)	C4—C3—H3	119.5
O1—C9—H9	108.1 (14)	C2—C3—H3	119.5
C11—C9—H9	110.6 (14)	F1—C15—C14	118.2 (2)
C10—C9—H9	104.4 (14)	F1—C15—C16	119.1 (2)
N1—C8—C7	112.81 (17)	C14—C15—C16	122.7 (2)
N1—C8—C10	100.47 (16)	C14—C13—C12	120.9 (2)
C7—C8—C10	116.89 (17)	C14—C13—H13	119.5
N1—C8—H8	108.8 (13)	C12—C13—H13	119.5
C7—C8—H8	108.5 (13)	N1—C19—H19A	109.5
C10—C8—H8	109.0 (13)	N1—C19—H19B	109.5
O2—C17—O3	123.2 (2)	H19A—C19—H19B	109.5
O2—C17—C10	124.1 (2)	N1—C19—H19C	109.5
O3—C17—C10	112.58 (19)	H19A—C19—H19C	109.5
C12—C11—C16	119.3 (2)	H19B—C19—H19C	109.5
C12—C11—C9	122.1 (2)	O3—C18—H18A	109.5
C16—C11—C9	118.6 (2)	O3—C18—H18B	109.5
C10—C1—S1	112.17 (15)	H18A—C18—H18B	109.5
C10—C1—H1B	112.2 (14)	O3—C18—H18C	109.5
S1—C1—H1B	103.7 (14)	H18A—C18—H18C	109.5
C10—C1—H1A	109.2 (15)	H18B—C18—H18C	109.5
S1—C1—H1A	108.2 (15)	C19—N1—C8	113.99 (19)
H1B—C1—H1A	111 (2)	C19—N1—O1	103.60 (17)
C15—C16—C11	119.1 (2)	C8—N1—O1	102.23 (15)
C15—C16—H16	120.4		
			
C6—C7—C2—C3	0.8 (3)	O1—C9—C11—C12	−22.1 (3)
C8—C7—C2—C3	178.1 (2)	C10—C9—C11—C12	98.5 (3)
C6—C7—C2—S1	−175.09 (16)	O1—C9—C11—C16	157.1 (2)
C8—C7—C2—S1	2.2 (3)	C10—C9—C11—C16	−82.3 (3)
C1—S1—C2—C7	−12.6 (2)	C17—C10—C1—S1	61.9 (2)
C1—S1—C2—C3	171.40 (18)	C8—C10—C1—S1	−63.9 (2)
N1—O1—C9—C11	130.75 (19)	C9—C10—C1—S1	−172.93 (15)
N1—O1—C9—C10	3.2 (2)	C2—S1—C1—C10	42.50 (19)
C17—C10—C9—O1	−146.89 (19)	C12—C11—C16—C15	−0.2 (3)
C1—C10—C9—O1	85.8 (2)	C9—C11—C16—C15	−179.4 (2)
C8—C10—C9—O1	−30.8 (2)	C2—C7—C6—C5	−0.9 (3)
C17—C10—C9—C11	89.4 (2)	C8—C7—C6—C5	−178.3 (2)
C1—C10—C9—C11	−37.9 (3)	C16—C11—C12—C13	−0.1 (4)
C8—C10—C9—C11	−154.55 (19)	C9—C11—C12—C13	179.1 (2)
C6—C7—C8—N1	−87.2 (2)	C3—C4—C5—C6	0.1 (4)
C2—C7—C8—N1	95.5 (2)	C7—C6—C5—C4	0.4 (3)
C6—C7—C8—C10	157.06 (19)	C5—C4—C3—C2	−0.1 (4)
C2—C7—C8—C10	−20.2 (3)	C7—C2—C3—C4	−0.3 (4)
C17—C10—C8—N1	162.34 (17)	S1—C2—C3—C4	175.88 (19)
C1—C10—C8—N1	−70.2 (2)	C13—C14—C15—F1	179.4 (2)
C9—C10—C8—N1	47.06 (19)	C13—C14—C15—C16	−0.2 (4)
C17—C10—C8—C7	−75.3 (2)	C11—C16—C15—F1	−179.3 (2)
C1—C10—C8—C7	52.1 (2)	C11—C16—C15—C14	0.3 (4)
C9—C10—C8—C7	169.45 (17)	C15—C14—C13—C12	−0.1 (4)
C18—O3—C17—O2	−1.6 (4)	C11—C12—C13—C14	0.2 (4)
C18—O3—C17—C10	176.2 (2)	C7—C8—N1—C19	77.3 (2)
C1—C10—C17—O2	−142.9 (2)	C10—C8—N1—C19	−157.50 (19)
C8—C10—C17—O2	−17.1 (3)	C7—C8—N1—O1	−171.62 (16)
C9—C10—C17—O2	90.9 (3)	C10—C8—N1—O1	−46.41 (19)
C1—C10—C17—O3	39.4 (3)	C9—O1—N1—C19	146.0 (2)
C8—C10—C17—O3	165.18 (19)	C9—O1—N1—C8	27.3 (2)
C9—C10—C17—O3	−86.8 (2)		

### Hydrogen-bond geometry (Å, º)

Cg3 and Cg4 are the centroids of rings C2–C7 and C11–C16, respectively.

**Table:**

*D*—H···*A*	*D*—H	H···*A*	*D*···*A*	*D*—H···*A*
C6—H6···*Cg*4^i^	0.93	2.75	3.479 (3)	136
C13—H13···*Cg*3^ii^	0.93	2.74	3.599 (3)	153

Symmetry codes: (i) *x*+1/2, −*y*+1/2, *z*−1/2; (ii) −*x*+1, −*y*, −*z*+2.

## References

[bb1] Bruker (2004). *APEX2*, *SAINT*, *XPREP* and *SADABS*. Bruker AXS Inc., Madison, Wisconsin, USA.

[bb2] Farrugia, L. J. (2012). *J. Appl. Cryst.* **45**, 849–854.

[bb3] Khan, M. N., Tahir, M. N., Khan, M. A., Khan, I. U. & Arshad, M. N. (2008*a*). *Acta Cryst.* E**64**, o730. 10.1107/S1600536808007150 PMC2960905 21202120

[bb4] Khan, M. N., Tahir, M. N., Khan, M. A., Khan, I. U. & Arshad, M. N. (2008*b*). *Acta Cryst.* E**64**, o1704. 10.1107/S1600536808024628 PMC2960694 21201693

[bb5] Sheldrick, G. M. (2008). *Acta Cryst.* A**64**, 112–122. 10.1107/S0108767307043930 18156677

[bb6] Spek, A. L. (2009). *Acta Cryst.* D**65**, 148–155. 10.1107/S090744490804362X PMC2631630 19171970

