# Supplementary figures and images for: Crystal structure of methyl 3-(3-fluoro­phen­yl)-1-methyl-1,3a,4,9b-tetra­hydro-3H-thio­chromeno[4,3-c]isoxazole-3a-carboxyl­ate
Source: Acta Crystallogr E Crystallogr Commun. 2015 Jul 25;71(Pt 8):o600–1. doi: 10.1107/S2056989015013651 (PMC4571418; doi:10.1107/S2056989015013651)

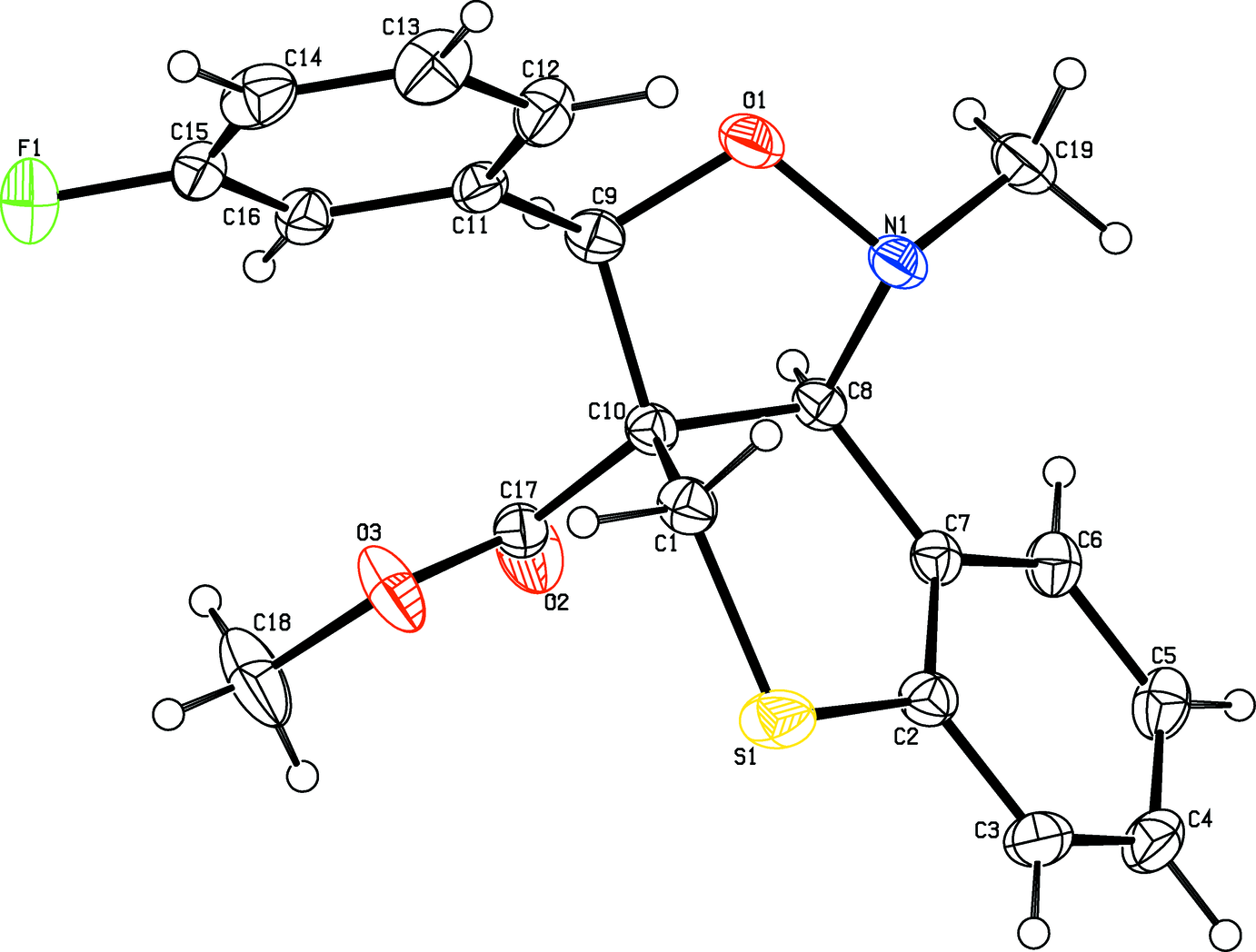

Supplement: Supplementary file 4 [file e-71-0o600-fig1.tif]
